# Diversity in changes of HRQoL over a 1-year period after radiotherapy in Norwegian breast cancer patients: results of cluster analyses

**DOI:** 10.1007/s11136-019-02127-7

**Published:** 2019-02-07

**Authors:** Magdalena Anna Lazarewicz, Dorota Wlodarczyk, Steinar Lundgren, Randi Johansen Reidunsdatter

**Affiliations:** 1 Department of Medical Psychology and Medical Communication, Medical University of Warsaw, Warsaw, Poland; 2 Department of Oncology, St. Olavs hospital, Trondheim University Hospital, Trondheim, Norway; 3 Department of Clinical and Molecular Medicine, Norwegian University of Science and Technology, Trondheim, Norway; 4 Department of Circulation and Medical Imaging, Norwegian University of Science and Technology, Trondheim, Norway

**Keywords:** Breast cancer, Oncology, Radiotherapy, Health-related quality of life, Cancer-related symptoms, Clusters of trajectories

## Abstract

**Purpose:**

The diversity in long-term changes in health-related quality of life (HRQoL) among breast cancer (BC) survivors is poorly understood. The aim of this study was to identify clusters of trajectories (subgroups of patients with similar patterns of changes) of selected HRQoL domains over a 1-year period after radiotherapy (RT) in BC patients.

**Methods:**

The group consisted of 250 BC patients referred for postoperative RT. Global quality of life (QoL), functions, and cancer-specific symptoms were assessed using the European Organisation for Research and Treatment of Cancer (EORTC) core Quality of Life Questionnaire (QLQ-C30) before starting RT, at completion of RT and 3, 6, and 12 months after RT. A hierarchical cluster analysis was used to identify possible trajectories of HRQoL domains.

**Results:**

Three distinct types of clusters of trajectories were identified for all outcome variables: Type 1 clusters encompassing the rather time-stable high-global QoL cluster, high-functioning clusters, and low-symptom clusters (44–98% of patients), Type 2 clusters with medium levels of HRQoL domains (8–49%), Type 3 clusters encompassing low-global QoL, low-functioning, and high-symptoms clusters (2–51%).

**Conclusions:**

Our results demonstrated a noticeable heterogeneity of changes in HRQoL domains after BC treatment. The findings support the importance of an accurate patient-reported HRQoL assessment as a routine element of BC survivors’ care. The pre-RT assessment of HRQoL alone allows to predict the course of HRQoL changes over the 1-year period after RT and the risk of “falling into” low functioning clusters.

**Electronic supplementary material:**

The online version of this article (10.1007/s11136-019-02127-7) contains supplementary material, which is available to authorized users.

## Background

During the last decade, health-related quality of life (HRQoL) and other patient-reported outcomes have acquired an increased attention in oncology, especially due to their predictive abilities for later events [1–3]. In breast cancer (BC), patients’ presurgical symptom profiles have shown an ability to predict HRQoL 2 years after treatment [1]. Psychological distress and fatigue two months after surgery are found to predict recurrence and survival [2]. Furthermore, a decline in physical functioning in the two 2 years after BC diagnosis may predict 10-year survival in older women [3]. The increased attention to HRQoL and other patient-reported outcomes is also due to findings revealing that physicians often underestimate patients’ symptoms and dysfunction [4], which could have a pronounced impact on the quality of health care patients receive.

BC survivors are today the largest group of cancer survivors [5]. Both the BC diagnosis and its treatment may have a disruptive impact on patients’ HRQoL [6–8]. Modern treatment includes surgery, usually in combination with one or more of the adjuvant treatments—radiotherapy (RT), chemotherapy, endocrine therapy, and immunotherapy (Herceptin)—which all may influence HRQoL both separately and in combinations. Chemotherapy is associated to fatigue, impaired cognition, insomnia, and pain [9–12] while RT alone may induce fatigue, breast symptoms, and arm/shoulder pain [12, 13]. BC patients may experience multiple concurrent symptom that strongly deteriorate their functioning and quality of life (QoL) [10, 11, 14]. Psychosocial morbidities and distress [14, 15] and diminished physical function [15] can persist for many years after treatment.

Longitudinal follow-up studies up to 12 years after BC treatment report global QoL and health equal to age-matched non-cancer controls [16] or age-matched general populations [17, 18]. However, some studies report worse cognitive functioning [9, 16] and poorer physical-, role-, emotional-, and social functioning and more symptoms in BC survivors compared to the reference groups [9, 17].

The development of patients’ post-treatment HRQoL is not sufficiently elaborated [7, 14, 19]. Some studies show that throughout the year following surgery patients’ HRQoL usually improves progressively [15–17] which seems to be very promising. However, this development may not apply to all patients. Recent evidence suggests that BC patients demonstrate more heterogeneity in the patterns of change in HRQoL over time following a BC diagnosis and treatment [1, 17] than it would appear on the base of average-level data. While most BC survivors experience little functional disturbances, some report persistent chronic disruption of normal functioning or initial functional disruption. Longitudinal studies estimating the pattern of functioning and symptoms are usually limited to the average-level data from the total sample [9, 11, 16–18, 20] or from subgroups defined by treatment modalities [12, 15], and thereby obscuring any individual patterns of changes. In BC populations, most studies have evaluated HRQoL during and after chemotherapy [11], and few studies have been designed to prospectively follow patients during and after RT with respect to symptoms, functioning, and QoL [12].

Inconclusive reports on the levels and trajectories of HRQoL domains in BC survivors, the dominance of studies limiting analysis to the average-level data, and the paucity of studies following BC patients’ HRQoL after RT makes further research on this topic relevant. We attempted to fill this gap by examining different patterns of changes in HRQoL over the first year following BC radiotherapy. The specific research questions were:

1. What are the clusters of trajectories of global QoL in this group of patients?
2. What are the clusters of trajectories of physical, emotional, cognitive, social and role functioning in this group?
3. What are the clusters of trajectories of selected cancer-specific symptoms (fatigue, pain, insomnia) in this group of patients?

## Methods

### Study group

The present study is a part of a larger longitudinal project investigating HRQoL in Norwegian BC survivors. All patients referred for postoperative RT (either alone or after chemotherapy) were consecutively invited to the study. Patients were included in the study if they (1) were referred for postoperative RT, (2) had no metastatic disease, (3) had no physical or psychological disorders that would interfere with participation, and (4) were able to speak and understand Norwegian.

Chemotherapy was administered routinely prior to RT as six anthracycline-based courses or four anthracycline-based courses followed by 12 weeks of taxane therapy. In local RT, 50 Gy was delivered to the breast/chest wall in 2 Gy/fraction, 5 days a week. Locoregional RT also included 46 Gy delivered to lymph nodes in the periclavicular region +/- the axillae.

### Study procedure

Out of 261 eligible patients, 250 (96%) agreed to participate. All assessments were conducted through an extended outpatient follow-up at the hospital, taking place before starting RT (T1), at completion of RT (T2), and 3, 6, and 12 months after RT (T3-T5). The follow-up-control included medical imaging of heart and lungs, clinical examinations, and QoL assessments by questionnaires. Oral and written study information was provided at the first consultation prior to RT. After inclusion, some patients omitted selected assessments due to long travel distances (*n* = 19), logistic problems (*n* = 49), a family incident (*n* = 1) or for unknown reasons (*n* = 4). Patients who developed metastatic diseases during follow-up, requested for exclusion or moved abroad, were excluded from the study (*n* = 10). The recruitment procedure and patients’ dropout are described in detail elsewhere [21]. The sample of patients who participated in all five assessments included 186 patients; however, in some cases, there was further missingness on different outcome variables leading to further decrease of sample sizes in the analyses of different QoL dimensions.

### Measures

At baseline (T1), sociodemographic data were collected from patients by self-report questionnaires. Clinical and treatment variables were registered by the oncologist. Clinical variables included stage of cancer (AJCC) and comorbidity. Treatment variables included type of surgery (conservative/radical), the extent of RT (local/locoregional) and the implementation of chemotherapy (Yes/No), endocrine therapy (Yes/No), and Herceptin (Yes/No).

Perceived symptoms and HRQoL indicators were measured by the European Organisation for Research and Treatment of Cancer (EORTC) core Quality of Life Questionnaire (QLQ-C30) [22]. The measures from QLQ-C30 addressed a global QoL subscale and five functional subscales: physical, cognitive, emotional, role, and social functioning during the previous week. The symptoms’ subscales included fatigue, pain, and insomnia during the past week. Response options ranged from 1 (very poor) to 7 (excellent) for global QoL and from 1 (not at all) to 4 (very much) for symptoms and functional subscales. All subscales scores were calculated according to the EORTC scoring manual [23] as the average score and transformed to a 0–100 scale, with higher scores indicating better global QoL and function, and more symptoms. Following recommendations from previously published evidence [24, 25], we interpreted functional subscales’ scores ≤ 60 and symptoms scores ≥ 40 as clinically significant impairment.

### Statistical methods

To examine if in the study sample there are subgroups with different courses of change (trajectories) of HRQoL domains over time, we used a hierarchical cluster analysis (as the method appropriate at exploratory stage of data analysis). Cluster analysis allows to classify cases into groups that are relatively homogeneous within themselves and relatively heterogeneous between each other [26]. Patients are grouped into clusters depending on the similarity of the trajectory of an analysed outcome variable. We used a squared Euclidean distance as a distance measure and complete linkage (the method creating the most consistent clusters with the smallest standard deviations) as a linkage method. The decisions regarding the final accepted solutions (number of clusters of trajectories) were made on the base of the agglomeration schedule (the increase in coefficients indicates that the clusters being combined at a given stage are more heterogeneous than previous combinations) and the dendrogram (a graphic presentation of the number of clusters and distance between them at consecutive stages of the process of calculation). The analyses were run separately for each outcome variable. In each analysis, results from all five measurements were included as cluster variables which finally demarcate trajectories (courses of change in the outcome variables over the five measurements). Final clusters were composed based on similar trajectories. Both two- and three-cluster solutions were acceptable (they brought an improvement of prediction accuracy with no such improvement for four-cluster solution). We decided to present results regarding three-cluster solutions as this allows for less information loss and more in-depth description of the sample than the two-cluster solution.

The repeated measures ANOVA with Bonferroni correction for post hoc analysis was conducted to compare mean scores on different measurements within clusters and its results are presented in Online Resource. For all statistical tests, significance level was set at 0.05.

### Missing data

A hierarchical cluster analysis includes cases with complete data, so the number of cases constituting the final clusters differed from the number of participants in the initial sample. In some cases, the analyzed sample sizes differed due to dissimilar missingness in different outcome variables. To analyze the nature of missingness, we used Little’s Mcar test which confirmed that missingness was completely at random (all *p*s > 0.05) for all aspects of quality of life except for social functioning (*p* = 0.037). Thus, we assumed that the obtained results are representative of the initial sample; however, the results regarding social functioning should be interpreted with caution.

## Results

### Demographic and medical characteristics of the sample

The demographic and medical characteristics of the initial sample are presented in Table 1.

**Table 1:** Demographic and medical characteristics of the initial sample (*N* = 250)

Demographic characteristics	*N* (%)	Medical characteristics	*N* (%)
Mean age (SD, range)	58.1 (9.85, 28–89)	AJCC	
Marital status		Stage 0	20 (8.0)
Living alone	59 (23.6)	Stage I	128 (51.2)
Married/cohabiting	189 (75.6)	Stage IIA	55 (22.0)
Missing	2 (0.80)	Stage IIB	23 (9.2)
Education		Stage III	24 (9.6)
Primary school (7–10 grade)	63 (25.2)	Comorbidity (yes)	72 (28.8)
Vocational (1–2 grades)	84 (33.6)	Surgery	
High school (2–4 grades)	35 (14.0)	Conservative	181 (72.4)
University, 3 years	31 (12.4)	Radical	69 (27.6)
University > 3 years	34 (13.6)	Radiotherapy	
Missing	3 (1.2)	Local	167 (66.8)
Employment		Locoregional	83 (33.2)
No	174 (69.6)	Chemotherapy (yes)	104 (41.6)
Yes	68 (27.2)	Neoadjuvant	24 (9.6)
Missing	8 (3.2)	Adjuvant	80 (32.0)
Total family income in NOK		Endocrine therapy (yes)	137 (54.8)
< 300 000	43 (17.2)	Herceptin (yes)	34 (13.6)
300,000–499,000	75 (30.0)		
> 500 000	105 (42.0%)		
Missing	27 (10.8)		

*SD* standard deviation, *NOK* Norwegian krone, *AJCC* a classification system developed by the American Joint Committee on Cancer for describing the extent of disease progression in cancer patients

The mean age of the patients was 58.1 years. Most of them were married or cohabiting and had secondary (14%) or higher (26%) education. The majority of women were not working at T1 and reported a family income between 300 and 500,000 NOK per year, which represents around the median income for women in Norway.

About half of the patients were diagnosed with stage I BC and most had no comorbidities. The majority of the sample underwent breast-conserving surgery and local RT. Postoperative chemotherapy had been given to 42% of the patients, endocrine therapy to 55%, and Herceptin to 14% of the patients.

### HRQoL measures

**Fig. 1:**
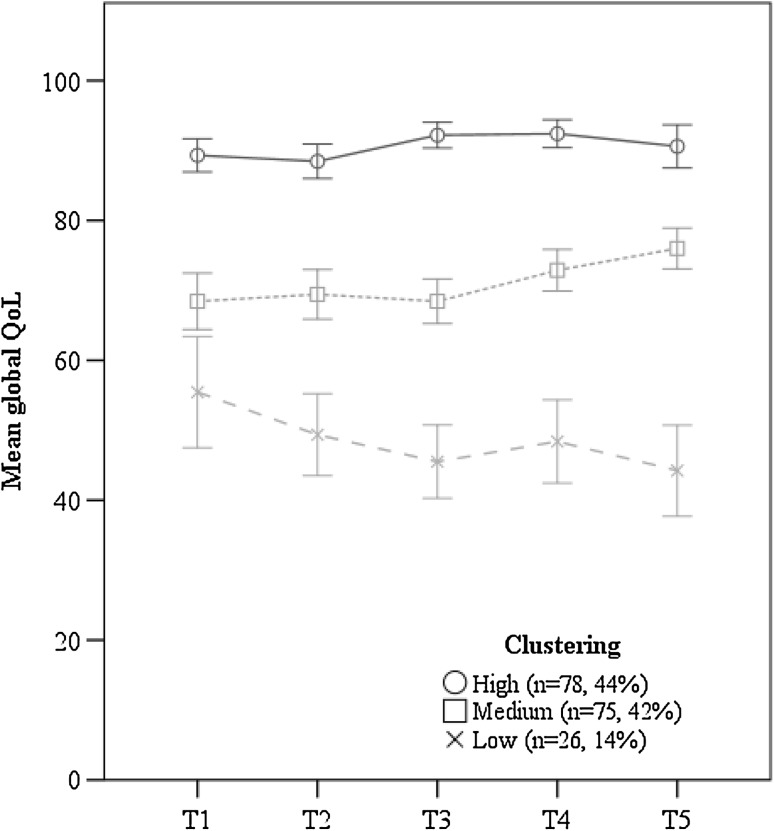
Clusters of trajectories of global Quality of Life during 1 year after radiotherapy (*N* = 179) in breast cancer patients. Measurements: T1—before starting RT, T2—at completion of RT, T3—3 months after RT, T4—6 months after RT, T5—12 months after RT. *RT* radiotherapy

**Fig. 2:**
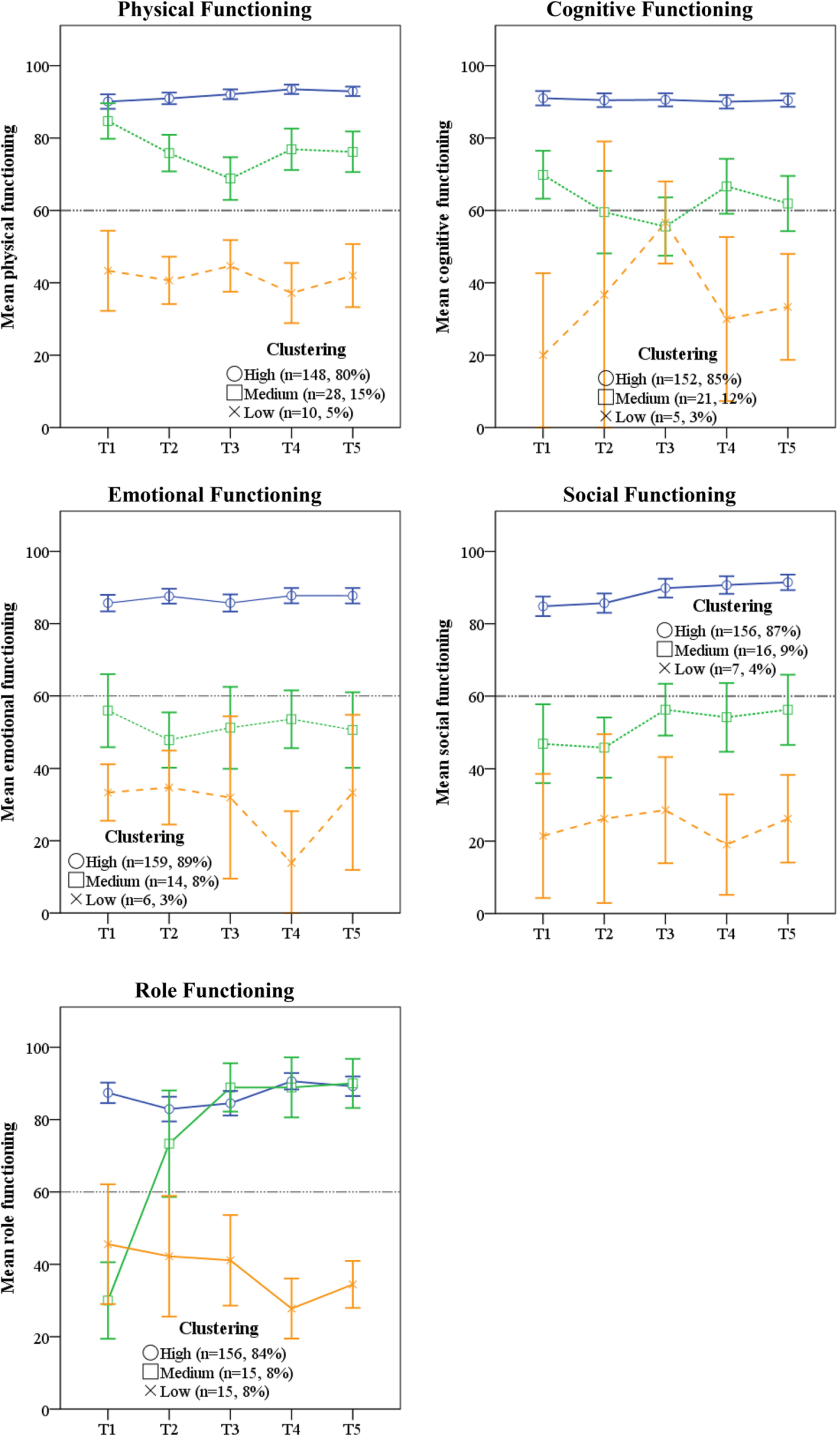
Clusters of trajectories of different aspects of breast cancer patient functioning during 1 year after radiotherapy. A 60 points cut-off point indicates a clinically significant impairment. Measurements: T1—before starting RT, T2—at completion of RT, T3—3 months after RT, T4—6 months after RT, T5—12 months after RT. *RT* radiotherapy

**Fig. 3:**
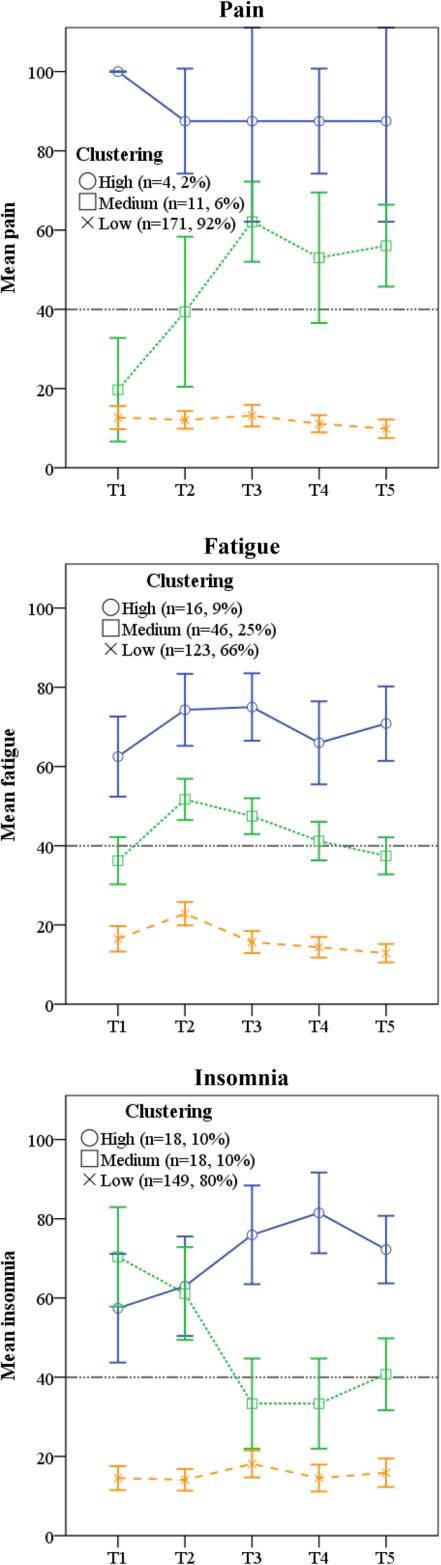
Clusters of trajectories of selected symptoms in breast cancer patients during 1 year after radiotherapy. A 40 points cut-off point indicates a clinically significant impairment. Measurements: T1—before starting RT, T2—at completion of RT, T3—3 months after RT, T4—6 months after RT, T5—12 months after RT. *RT* radiotherapy

The results showing the courses of trajectories together with percentages of patients belonging to them are presented in Figs. 1, 2 and 3. Each trajectory presents means with confidence intervals. Additionally, for functional and symptom subscales (Figs. 2, 3), cut-off points for clinically significant impairment are shown.

#### Clusters of trajectories of global QoL

The three distinct clusters of trajectories of global QoL can be described as “high,” “medium,” and “low” clusters, with “high” and “medium” clusters being equally common (Fig. 1, see detailed statistics in Online Resource Table 1a). The “high” cluster showed a rather stable QoL over the T1–T5 period with mean scores (SD) ranging from 89(11) to 92(9), with a small but significant increase between T1 and T2 to T3 and T4. The “medium” cluster showed a stable medium QoL between T1 and T3, and a significant increase thereafter, with mean scores (SD) ranging from 68(18) at T1 to 76(13) at T5. The less common “low” cluster showed a significant decrease from T1 to T3, T4 and T5, with mean scores (SD) from 56(20) at T1 to 44(16) at T5.

#### Clusters of trajectories of BC survivors functioning

Clusters of trajectories of different aspects of functioning are presented in Fig. 2 (see detailed statistics in Online Resource Table 1b).

The “high-functioning” clusters were the dominant groups representing 80–89% of the patients reporting good and stable functioning at all five assessments. The level of functioning was best in the physical domain with mean scores (SD) between 90(12) and 94(8), followed by cognitive functioning with mean scores between 90(11) and 91(12). The level of emotional functioning slightly fluctuated from 85(15) to 88(14), social functioning increased significantly from 85(17) at T1 to 92(14) at T5, while role functioning increased significantly from 83(22) at T2 to 91(14) at T4.

The “medium-functioning” clusters represented 8–15% of the patients and showed more fluctuating courses than the “high-functioning” clusters. Physical function decreased significantly from 85(13) at T1 to 68(15) at T3, and thereafter increased significantly reaching the level of 76(14) at T4 and T5. Cognitive function showed similar pattern with a significant drop from 70(15) at T1 to 56(18) at T3, following by a small improvement to 62(17) at T5. Emotional function had a slightly fluctuating pattern from 55(17) at T1 to 51(18) at T5, while social functioning showed a small but significant increase from 47(20) at T1 to 56(18) at T5. The “medium role-functioning” cluster appeared with a considerable different course than the other functioning clusters, starting very low at 30(19) at T1, increased significantly to 73(27) at T2, and further to 89(12) at T3 and 90(12) at T5.

The “low-functioning” groups represented 3–5% of the patients. Physical function showed a slightly fluctuating course varying between 44(10) at T3 to 37(12) at T4. Emotional function remained stable between 33(8) at T1 and 32 (21) at T3, then decreased to 14(13) at T4, and thereafter increased back to 33(21) at T5. Cognitive function showed a different pattern by increasing significantly from 20(18) at T1 to 56(9) at T3 and thereafter dropped significantly to 33(12) at T5, while role function decreased from 45(30) at T1 to 28(12) at T4 and thereafter slightly increased to 33(12). The low social functioning cluster slightly fluctuated between 21(19) and 29(16) during the 5 assessments.

#### Clusters of trajectories of cancer-specific symptoms

Among the symptom subscales (Fig. 3, see detailed statistics in Online Resource Table 1c), the “low-symptom” clusters represented the majority (67–92%) of patients and showed relatively stable low levels of symptoms during the follow-up. The mean (SD) level of fatigue was 17(18) at T1, increased slightly during RT to 23(17) at T2, and thereafter gradually decreased to 13(13) at T5. The “low-pain” cluster showed a rather stable low level of pain over the T1-T3 period, with a significant decrease of this symptom between T3 and T5 and mean scores (SD) ranging from 10(16) to 13(18). The level of insomnia stayed rather stable ranging from 14(17) to 18(21), with a slight but significant increase from T2 to T3.

The “medium-symptom” clusters represented 6–25% of the patients and acted differently in different symptoms. The “medium-fatigue” cluster showed a significant increase of fatigue from T1 to T2, followed by a steady decrease from T2 to T4 and T5, with mean scores (SD) ranging from 36(20) to 52(18). Pain showed a dramatic increase from T1 to T3, stabilizing afterwards at the medium (but clinically significant) level, with mean scores (SD) ranging from 20(19) to 62(15). The “medium insomnia” cluster showed the pattern opposite to pain: it stared at a high and clinically significant level of 70(25) at T1 and 61(23) at T2. Thereafter, the sleep problems decreased to a level of 33(23) at T3 and T4, and ended at a level of 41(18) at T5.

The “high-symptom” clusters represented 2–10% of the patients. The high fatigue and high pain clusters remained rather stable, with mean scores (SD) ranging from 62.5(19.0) to 75.0(16.0) and 88(8) to 100(0), respectively. The high insomnia cluster showed a significant steady increase of insomnia from T1 to T4, followed by a decrease between T4 and T5 (*p* = 0.056), with mean scores (SD) ranging from 57(28) to 82(21).

## Discussion

The results of cluster analyses revealed three clusters of trajectories for all outcome variables and allowed to group these clusters into three types:

- Type 1 encompassing high global QoL cluster, high function (emotional, physical, cognitive, social, and role function) clusters, and low symptom (fatigue, insomnia, and pain) clusters;
- Type 2 encompassing medium global QoL cluster, medium function (emotional, physical, cognitive, social, and role) clusters, and medium symptoms (fatigue, insomnia, and pain) clusters;
- Type 3 encompassing low global QoL cluster, low function (emotional, physical, cognitive, social, and role) clusters, and high symptoms (fatigue, insomnia, and pain) clusters.

For global QoL, two similarly numerous groups emerged: one reporting quite high, slightly increasing global QoL, and the other reporting high and stable QoL. These trajectories are similar to those revealed in previous studies limiting the analysis to average-level data [16, 17, 20]. However, the third less numerous group demonstrated significantly lower and further decreasing global QoL over the 1-year follow-up. These diverse courses of global QoL after BC treatment show that this domain may be more complex than it was previously presented in the literature [15, 16].

A somewhat similar pattern of heterogeneity in trajectories was observed for the physical-, emotional-, social-, and cognitive-function scales, with one dominating and two less numerous clusters.

As for physical functioning, apart from the two clusters with very high and high scores, we observed one group (5%) with scores which are classified as clinically significant impairment (cut-off point at 60 points) [25]. This group of patients should receive special attention from health professionals as it was proved that patients who experienced a decline in physical functioning without recovery within 2 years are at risk of functional decline and early mortality [4].

In case of emotional, social, and cognitive functioning, patients belonging to the clusters with the lowest scores (3%, 4% and 3%, respectively) demonstrated clinically significant impairment, whereas those who belonged to the medium clusters (8%, 9%, and 12%, respectively) scored close to the level of the cut-off point. Importantly, it was proved that only a high level of emotional functioning (> 83 points) was a predictor of overall survival [3]. In the data presented by others [16, 20], trajectories for emotional functioning resembled only those changes which in our data were reported for the cluster scoring the highest. Thus, we got insight into the diversity of functioning trajectories which has not been presented in the literature yet.

For social functioning, the “medium” and “low” cluster represented 11% of patients with significantly diminished functioning (< 56 and < 29 points, accordingly). Social support tailored to the patient’s individual needs has a favourable impact on both the mental and physical QoL [27]. Identifying BC survivors lacking social support may enable them to be guided to appropriate support networks and programs.

As for role functioning, we reported that 16% (8% +8%) of patients demonstrated clinically significant impairment before RT, but three months after RT half of this group achieved as high level as the best-functioning cluster. The remaining half of the group presented limitations in work, leisure time, or other daily activities with a tendency to deterioration. Recognizing specific factors responsible for improvement or deterioration over time would be critical for further care delivered to these patients [28].

Moreover, six months after treatment seems to be a critical period for patients within the lower clusters of all the above-mentioned functioning domains. A functioning decrease from three to six months follow-up could be due to an experience of being “left alone” as this is the first period in the treatment course without any planned contact with health professionals [28, 29].

In the cancer-specific symptoms, we also identified one dominating cluster with low symptom scores and relatively high stability over time. However, in the case of pain, there was a small group (6%) which started with low intensity but directly after RT demonstrated clinically significant pain. Pain in the breast due to acute soreness of the skin and subsequent swelling is the most common acute side-effect from RT [13] and is, therefore, most likely to emerge immediately after RT. We measured general pain based on two following items “have you had pain?” and “did pain interfere with your daily activities?” Thus, the reported pain could be ascribed to other causes like comorbidities or problems related to the surgery, but those patients are more likely belonging to the very small high-symptom cluster, as this cluster was at a stable high level during the follow-up.

In the case of insomnia, there was a group (20%) which before and directly after RT revealed clinically significant sleep disturbance, but later half of them dropped to around the cut-off point, while the other half experienced substantial sleep disturbances. Poor sleep is strongly associated to decreased HRQoL [11] and is, therefore, essential to be examined in the follow-up procedures.

Importantly, there was a relatively numerous group of patients (34%) experiencing clinically significant fatigue at most assessments. As a low level of fatigue (0–22 points) is a significant predictor of recurrence-free survival in BC patients [3], patients with high levels of fatigue also require special attention from health professionals. Regardless of fitting in the cluster, all patients demonstrated an increase in fatigue directly after RT. This pattern is similarly observed in several studies [12] and is likely caused by the acute and temporarily elevated inflammatory response due to RT [30].

### Study limitations

The study has some limitations. First, our findings may to some extent reflect the characteristics of the study group; half of the BC patients were diagnosed with stage I BC and the majority had not received chemotherapy, had no comorbidity and underwent breast conserving surgery. Such factors as less advanced stage of BC, lack of axillary dissection or breast-conserving surgery were proved to be associated with better HRQoL [31]. This may be responsible not only for very high scores in the high clusters but also for the relatively small sizes of the remaining clusters. Thus, further research encompassing patients with more varied health conditions is necessary. Second, the main follow-up study was designed for exploring HRQoL in BC patients receiving RT (i.e., first assessment before RT). However, due to the explorative design, around 40% of the cohort had also received chemotherapy before RT. Thus, our outcome measures might be influenced by chemotherapy as well. Third, the observations presented in this study included only a 1-year post-RT perspective. It would be valuable to extend the study for a longer period. Furthermore, our study focused only on analysis and description of possible heterogeneity in trajectories of selected domains of BC survivors’ HRQoL. Further analyses should concentrate on searching for factors which determine their assignment to the particular cluster (high versus medium versus low HRQoL). Such results would have a great clinical value, allowing the early recognition of BC patients at risk of HRQoL deterioration.

## Conclusions

The results of this study complement previous research by demonstrating clusters of patients with poorer HRQoL (Type 3 clusters with lower global QoL, lower functioning and higher symptoms) in comparison to the majority who are having significantly better outcomes (Type 1 and Type 2). These BC survivors are at risk of further worsening of HRQoL, cancer recurrence, or higher mortality [3, 4] and may have specific needs that require supportive attention from health professionals. Future health care for them can be planned and individually adjusted on the basis of the early (T1) assessment of HRQoL. Thus, our results highlight the importance of an accurate patient-reported HRQoL assessment as a routine element of BC patients’ care [32–34]. This need is especially true considering the evidence that physicians tend to underestimate patients’ poor HRQoL in comparison to reports by the patients themselves [3]. Early HRQoL assessment and selection of BC patients at risk of HRQoL deterioration would have a great clinical value and a pronounced impact on the quality of health care received by patients.

## Electronic supplementary material

Below is the link to the electronic supplementary material.

Supplementary material 1 (DOCX 42 KB)
