# Supplementary material for: Diversity in changes of HRQoL over a 1-year period after radiotherapy in Norwegian breast cancer patients: results of cluster analyses
Source: Qual Life Res. 2019 Feb 7;28(6):1521–30. doi: 10.1007/s11136-019-02127-7 (PMC6522443; doi:10.1007/s11136-019-02127-7)
Supplement: Supplementary file 1 — Supplementary material 1 (DOCX 42 KB) [file 11136_2019_2127_MOESM1_ESM.docx]

Online Resource.

Table 1a. Descriptive Statistics and Repeated Measures ANOVAs with Bonferroni Correction for Post Hoc Analysis for Global Quality of Life Clusters of Breast Cancer Patients

|  | T1 | T2 | T3 | T4 | T5 | F(*p*) | Significant differences* |
| --- | --- | --- | --- | --- | --- | --- | --- |
| Cluster | M(SD) | M(SD) | M(SD) | M(SD) | M(SD) |  |  |
| High (*n*=78) | 89.3(10.5) | 88.5(10.9) | 92.2(8.20) | 92.4(8.83) | 90.6(13.7) | 2.71(.036)^a^ | *no sig. differences* |
| Medium (*n*=75) | 68.5(17.5) | 69.4(15.5) | 68.4(13.9) | 72.9(12.9) | 76.0(12.7) | 4.31(.002) | T1,T3<T5 |
| Low (*n*=26) | 55.4(19.7) | 49.4(14.5) | 45.5(13.0) | 48.4(14.7) | 44.2(16.1) | 1.75(.175) |  |

Note. T1 – assessment before starting radiotherapy; T2 – assessment at completion of radiotherapy; T3 – assessment 3 months after radiotherapy; T4 – assessment 6 months after radiotherapy; T5 – assessment 12 months after radiotherapy.

^a^ A repeated-measures ANOVA with Huynh-Feldt correction

*The mean difference is significant at the <.05 level.

Table 1b. Descriptive Statistics and Repeated Measures ANOVAs with Bonferroni Correction for Post Hoc Analysis for Function Clusters of Breast Cancer Patients

|  | T1 | T2 | T3 | T4 | T5 | F(*p*) | Significant differences* |
| --- | --- | --- | --- | --- | --- | --- | --- |
| Cluster | M(SD) | M(SD) | M(SD) | M(SD) | M(SD) |  |  |
| Physical functioning | | | | | | | |
| High (*n*=148) | 90.1(12.3) | 91.0(9.78) | 92.1(8.32) | 93.5(7.99) | 92.9(8.07) | 6.98(<.001)^a^ | T1<T4,T5; T2<T4 |
| Medium (*n*=28) | 84.7(12.6) | 75.8(13.0) | 68.8(15.2) | 76.9(14.8) | 76.2(14.5) | 5.96(<.001) | T1>T2,T3 |
| Low (*n*=10) | 43.3(15.5) | 40.7(9.14) | 44.7(9.96) | 37.2(11.6) | 42.0(12.2) | 1.25(.307) |  |
| Emotional functioning | | | | | | | |
| High (*n*=159) | 85.7(14.6) | 87.6(13.1) | 85.7(15.1) | 87.7(13.5) | 87.7(13.6) | 1.81(.130)^a^ |  |
| Medium (*n*=14) | 56.0(17.4) | 47.8(13.2) | 51.2(19.6) | 53.6(13.8) | 50.6(18.0) | .490(.743) |  |
| Low (*n*=6) | 33.3(7.45) | 34.7(9.74) | 31.9(21.4) | 13.9(13.6) | 33.3(20.4) | 2.24(.101) |  |
| Cognitive functioning | | | | | | | |
| High (*n*=152) | 91.0(12.4) | 90.5(11.8) | 90.6(11.3) | 90.0(11.7) | 90.5(11.4) | .270(.897)^a^ |  |
| Medium (*n*=21) | 69.8(14.5) | 59.5(25.0) | 55.6(17.7) | 66.7(16.7) | 61.9(16.8) | 2.08(.091) |  |
| Low (*n*=5) | 20.0(18.3) | 36.7(34.2) | 56.7(9.13) | 30.0(18.3) | 33.3(11.8) | 2.20(.189)^b^ |  |
| Social functioning | | | | | | | |
| High (*n*=156) | 84.8(17.1) | 85.7(16.9) | 89.9(16.5) | 90.7(15.4) | 91.5(13.6) | 9.59(<.001)^a^ | T1<T3,T4,T5; T2<T4,T5 |
| Medium (*n*=16) | 46.9(20.4) | 45.8(15.5) | 56.3(13.4) | 54.2(17.7) | 56.2(18.1) | 1.42(.239) |  |
| Low (*n*=7) | 21.4(18.5) | 26.2(25.2) | 28.6(15.9) | 19.0(15.0) | 26.2(13.1) | .345(845) |  |
| Role functioning | | | | | | | |
| High (*n*=156) | 87.4(17.9) | 82.9(21.5) | 84.5(21.5) | 90.6(14.3) | 89.2(17.1) | 8.33(<.001) | T2<T1,T4,T5; T3<T4 |
| Medium (*n*=15) | 30.0(19.1) | 73.3(26.6) | 88.9(12.1) | 88.9(15.0) | 90.0(12.3) | 29.6(<.001) | T1<T2,T3,T4,T5 |
| Low (*n*=15) | 45.6(30.0) | 42.2(30.1) | 41.1(22.6) | 27.8(15.0) | 34.4(11.7) | 2.56(.089)^b^ |  |

Note. T1 – assessment before starting radiotherapy; T2 – assessment at completion of radiotherapy; T3 – assessment 3 months after radiotherapy; T4 – assessment 6 months after radiotherapy; T5 – assessment 12 months after radiotherapy. ^a^ A repeated-measures ANOVA with Huynh-Feldt correction; ^b^ A repeated-measures ANOVA with Greenhouse-Geisser correction; *The mean difference is significant at the <.05 level.

Table 1c. Descriptive Statistics and Repeated Measures ANOVAs with Bonferroni Correction for Post Hoc Analysis for Symptom Clusters of Breast Cancer Patients

|  | T1 | T2 | T3 | T4 | T5 | F(p) | Significant differences* |
| --- | --- | --- | --- | --- | --- | --- | --- |
| Cluster | M(SD) | M(SD) | M(SD) | M(SD) | M(SD) |  |  |
| Pain | | | | | | | |
| High (*n*=4) | 100.0(.0) | 87.5(8.33) | 87.5(16.0) | 87.5(8.33) | 87.5(16.0) | 1.08(.409) |  |
| Medium (*n*=11) | 19.7(19.5) | 29.4(28.2) | 62.1(15.1) | 53.0(24.5) | 56.1(15.4) | 7.25(<.001) | T1<T3,T4,T5 |
| Low (*n*=171) | 12.7(19.4) | 12.1(14.8) | 13.2(18.1) | 11.1(14.3) | 9.84(15.6) | 1.62(.175)^a^ |  |
| Fatigue | | | | | | | |
| High (*n*=16) | 62.5(19.0) | 74.3(17.1) | 75.0(16.0) | 66.0(19.7) | 70.8(17.6) | 1.45(.228) |  |
| Medium (*n*=46) | 36.2(20.0) | 51.7(17.6) | 47.5(15.3) | 41.2(16.4) | 37.4(15.8) | 6.73(<.001)^a^ | T1<T2; T5<T2,T3 |
| Low (*n*=123) | 16.5(18.0) | 22.9(16.5) | 15.7(15.5) | 14.4(14.7) | 12.9(13.0) | 11.1(<.001)^a^ | T2>T1,T3,T4,T5 |
| Insomnia | | | | | | | |
| High (*n*=18) | 57.4(27.5) | 63.0(25.2) | 75.9(25.0) | 81.5(20.5) | 72.2(17.1) | 3.65(.009) | T1<T4 |
| Medium (*n*=18) | 70.3(25.3) | 61.1(23.6) | 33.3(22.9) | 33.3(22.9) | 40.7(18.3) | 11.0(<.001) | T1>T3,T4,T5; T2>T3,T4 |
| Low (*n*=149) | 14.5(18.7) | 14.1(17.0) | 18.1(21.0) | 14.5(21.0) | 15.9(22.1) | 1.45(.220)^a^ |  |

Note. T1 – assessment before starting radiotherapy; T2 – assessment at completion of radiotherapy; T3 – assessment 3 months after radiotherapy; T4 – assessment 6 months after radiotherapy; T5 – assessment 12 months after radiotherapy.

^a^ A repeated-measures ANOVA with Huynh-Feldt correction

*The mean difference is significant at the <.05 level.
